# Interstitial Score and Concentrations of IL-4Rα, PAR-2, and MMP-7 in Bronchoalveolar Lavage Fluid Could Be Useful Markers for Distinguishing Idiopathic Interstitial Pneumonias

**DOI:** 10.3390/diagnostics11040693

**Published:** 2021-04-13

**Authors:** Magdalena Bruzova, Martina Pavlova, Radoslav Matej, Martina Sterclova, Martina Vasakova

**Affiliations:** 1 Department of Pathology and Molecular Medicine, 3rd Faculty of Medicine, Charles University and Thomayer University Hospital, Videnska 800, 140 59 Prague, Czech Republic; magdalena.smetakova@ftn.cz (M.B.); radoslav.matej@ftn.cz (R.M.); 2 Department of Respiratory Medicine, University Hospital Pilsen, Edvarda Benese 1128/13, 305 99 Pilsen, Czech Republic; kolbekovam@tiscali.cz; 3 Department of Pathology, 3rd Faculty of Medicine, Charles University and University Hospital Kralovske Vinohrady, Srobarova 1150/50, 100 34 Prague, Czech Republic; 4 Department of Pathology, 1st Faculty of Medicine, Charles University and General University Hospital, Studnickova 2, 128 00 Prague, Czech Republic; 5 Department of Respiratory Medicine, 1st Faculty of Medicine, Charles University and Thomayer University Hospital, Videnska 800, 140 59 Prague, Czech Republic; martina.vasakova@ftn.cz

**Keywords:** tumor necrosis factor-α, interleukin-4 receptor α, proteinase-activated receptor-2, matrix metalloproteinase-7, B cell-activating factor, interstitial score, idiopathic interstitial pneumonias, bronchoalveolar lavage fluid

## Abstract

Idiopathic interstitial pneumonia (IIP) entails a variable group of lung diseases of unknown etiology. Idiopathic pulmonary fibrosis, nonspecific interstitial pneumonia, interstitial lung diseases related to connective tissue disease (CTD-ILD), and hypersensitivity pneumonitis (HP) can manifest with similar clinical, radiological, and histopathological features. In a differential diagnosis, biomarkers can play a significant role. We assume that levels of specific cyto- or chemokines or their receptors can signal pathogenetic processes in the lungs. Eighty patients with different types of idiopathic interstitial pneumonia were enrolled in this study. Cell counts and concentrations of tumor necrosis factor (TNF)-α, interleukin-4 receptor α, proteinase-activated receptor (PAR)-2, matrix metalloproteinase (MMP)-7, and B cell-activating factor were measured in bronchoalveolar lavage fluid using commercial ELISA kits. High resolution computer tomography results were evaluated using alveolar and interstitial (IS) score scales. Levels of TNF-α were significantly higher in HP compared to fibrosing IIP (*p* < 0.0001) and CTD-ILD (*p* = 0.0381). Concentrations of IL-4Rα, PAR-2, and MMP-7 were positively correlated with IS (*p* = 0.0009; *p* = 0.0256; *p* = 0.0015, respectively). Since TNF-α plays a major role in inflammation, our results suggest that HP is predominantly an inflammatory disease. From the positive correlation with IS we believe that IL-4Rα, PAR-2, and MMP-7 could serve as fibroproliferative biomarkers in differential diagnosis of IIP.

## 1. Introduction

Idiopathic interstitial pneumonia (IIP) entails a variable group of lung diseases of unknown etiology. Different prognoses and therapies are based on the progression of fibrosis and inflammation within the parenchyma of the lungs [1]. The most common type and with the worst prognosis is idiopathic pulmonary fibrosis (IPF). The histological pattern of usual interstitial pneumonia (UIP) is a morphological background of IPF; however, this pattern can be seen in other IIPs. IPF is usually diagnosed after other identifiable causes of fibrosis have been excluded [2]. A less common type of IIPs is nonspecific interstitial pneumonia (NSIP), which is the most common histopathological pattern in interstitial lung disease(s) (ILD) related to CTD (CTD-ILD) and can be associated with significant mortality [3,4]. Hypersensitivity pneumonitis (HP) is caused by repetitive exposure to mainly organic inhalation antigens and has a characteristic histopathological pattern that is different in the fibrotic and nonfibrotic forms [5,6]. The similarities and common clinical, radiological, and histopathological features of the above-mentioned diseases complicate the diagnosis, treatment, and prognosis of these diseases. A multidisciplinary approach is crucial for making the correct IIP diagnosis and a combination of clinical, immunological, radiological, and histopathological investigations are essential for establishing both the diagnosis and treatment [7]. Biomarkers can play a significant role in making a diagnosis. There are many different biomarkers used in making a IIP differential diagnosis; some are used in routine practice while others have been used in experimental studies with varying degrees of success, i.e., sensitivity and specificity [2,8,9,10,11,12,13,14,15]. 

Alveolar macrophages (AMs), together with interstitial macrophages, are present in the lungs during homeostasis [16]. Under pathologic conditions, AMs express different chemokines and cytokines, which are involved in inflammation and fibrosis [17]. In IIPs, AMs are the key cells responsible for inducing fibroproliferative healing [18]. 

Tumor necrosis factor (TNF)-α is one of the cytokines produced by AMs in human lungs [18]. This pro-inflammatory cytokine is released right after trauma or infection and plays a pivotal role in orchestrating the production of the pro-inflammatory cytokine cascade, which is why it is also called the “master regulator” [19].

Proteinase-activated receptor (PAR)-2 is highly expressed on the epithelial cells and macrophages in the respiratory tract [20]. It is involved in proinflammatory and allergic responses since its activators are associated with inflammation and immune cell activity [21]. However, PAR-2 also has cytoprotective as well as anti-inflammatory functions [20].

Matrix metalloproteinase (MMP)-7 (also called matrilysin 1) is expressed by lung epithelial cells, mononuclear phagocytes, and fibrocytes [22]. According to previous studies, MMP-7 seems to be a potential biomarker for the differential diagnosis of IPF and a prognostic marker of ongoing pathology [8,9,23,24,25]. 

B cell-activating factor (BAFF) is a member of the TNF family and functions as an important regulator of the B cell immunological response [26]. BAFF has been detected in high levels in the blood and tissues of patients with autoimmune rheumatic diseases [26] and in plasma of patients with IPF [27].

In different IIPs, fibroproliferative pathways can lead to the same result; thus, there might be molecular patterns that can differentiate IIPs with similar radiologic and histopathologic findings. We hypothesize that the levels of cytokines, chemokines, and their receptors in bronchoalveolar lavage fluid (BALF) may reflect pathogenetic processes in the lungs and may correlate with specific HRCT scores for specific IIP. Therefore, evaluating those biomarkers in BALF at the time of diagnosis could provide support for a particular diagnosis, without the need for a surgical lung biopsy (SLB); they might also be used as prognostic biomarkers for predicting fibroproliferative healing in IIPs. Additionally, these biomarkers might be helpful in finding a treatment that can be tailored to each patient, regardless of the IIP, and be used to track the pharmacological effect of the treatment.

## 2. Materials and Methods

### 2.1. Patients

Eighty patients with different types of IIP (24 fibrosing IIP (fIIP), 20 CTD-ILD, and 36 HPs) were enrolled in the study. In the fIIP group, both IPF (*n* = 16) and NSIP (*n* = 8) patients were included. The HP group included nonfibrotic (*n* = 30) as well as fibrotic (*n* = 6) HP patients. The patients were naive relative to immunosuppressive drugs or any specific treatment for IIPs at the time of entering the study and underwent a set of investigations (described below). The assessment of an IIP diagnosis was based on current diagnostic guidelines and an MDT discussion [5,28]. All patients signed informed consent before entering the study. The study protocol was approved by the Central Ethics Committee of the Institute for Clinical and Experimental Medicine and Thomayer University Hospital, Prague, Czech Republic (protocol no. G-16-10-01; 18 October 2016). The study was conducted in accordance with the Declaration of Helsinki. Apart from that, the privacy of patients was fully respected during statistical analysis.

### 2.2. Lung Function Testing

At the time of diagnosis, all patients underwent a functional investigation of the lungs, i.e., spirometry and a diffusion lung capacity measurement for carbon monoxide (DL_CO_). Spirometry was performed using a ZAN 100 Flowhandy II (ZAN Messgerate, GmbH, Oberthulba, Germany), and DL_CO_ was measured using a Diffustik (Geratherm Respiratory GmbH, Germany).

### 2.3. High Resolution Computer Tomography (HRCT)

An HRCT investigation was performed at the time of diagnosis, and a modified HRCT visual scoring system, based on the IPF HRCT description system of Gay et al. [29], was used [30]. Based on this modified scoring system, the HRCT results were evaluated using alveolar (AS) and interstitial (IS) scores, where AS represents the extent of ground glass opacities and IS the extent of reticulations and/or honey-combing. AS and IS were evaluated by trained respiratory specialists using the alveolar and interstitial visual scoring system with the goal of quantifying the extent of the inflammatory and fibrotic changes [30]. AS and IS were assessed at four levels: aortic arch, tracheal bifurcation, maximal width of the right ventricle, and the right side of the diaphragm. After being scored separately, the mean values, ranging from 0 to 5, were counted (Table 1). The values are stated in percentages and describe the extent of changes, considering the disease stable for value 0 and 1 and progressive for higher values.

### 2.4. Bronchoscopy with Bronchoalveolar Lavage and Transbronchial Biopsy

Fiberoptic bronchoscopy, under local anesthesia, and bronchoalveolar lavage (BAL) were also performed at the time of diagnosis. The bronchoscope was wedged in the segmental part of the middle lobe, where four 50 mL fractions (i.e., 200 mL in total) of lukewarm saline were instilled. The fluid was retrieved using syringe suction, put into a sterile container and stored for further analysis. The BAL procedure was considered valid if the fluid recovery exceeded 25 mL (50%) per instilled fraction, and there was an absence of a significant admixture of polymorphic bronchial epithelial cells (no more than 10%). After BAL procedure, a standard transbronchial biopsy or transbronchial cryobiopsy was taken during bronchoscopy for diagnostic purposes and tissue fragments were histopathologically investigated following a standardized diagnostic protocol.

### 2.5. Laboratory Evaluation

Ten mL of the retrieved BAL fluid (BALF) was used for a cytologic investigation after cytocentrifugation and Giemsa-Romanowsky staining. The total count of cells was not routinely determined, the proportions of each cell type were calculated from the same predetermined numbers of cells (adapted from [31]). Another 10 mL of the retrieved BALF was centrifuged, and the supernatant was stored at −80 °C until analysis. Concentrations of TNF-α, IL-4Rα, PAR-2, MMP-7, and BAFF were measured using the ELISA method according to manufacturer’s instructions (Cloud-Clone Corp, Houston, USA). The concentrations of analytes were determined using a standard curve.

### 2.6. Statistics

The basic statistical characteristics, i.e., mean values and standard deviations were calculated for the quantitative variables (age, IS and AS, BALF differential cell counts and analyte concentrations, forced vital capacity (FVC), and diffusing capacity for carbon monoxide (DL_CO_)). Frequencies were used for the description of discrete variables (gender, smoking status). Statistical analyses were performed using GraphPad Prism 5 (La Jolla, CA, USA). Spearman’s rank-order correlation was used to assess the correlation between IS and AS relative to the concentrations of analytes in the BALF, lung functions, and BALF differential cell counts within the IIP subgroups (all three subgroups together). The Kruskal–Wallis ANOVA test with Dunn’s multiple comparison test, at a significance level of 0.05, was used for comparisons of analyte concentrations in BALF among fIIP, CTD-ILD, and HP subgroups. For significant differences between individual diagnoses, the Mann–Whitney two-tailed t-test with a 95% confidence interval was used. 

## 3. Results

The demographic data, lung functions, and BALF cell counts of patients with different types of IIPs are presented in Table 2. 

**Figure 1:**
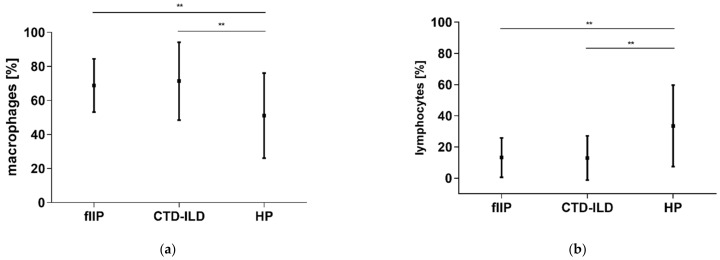
Distribution of statistically significant differential cell counts among all pathologies tested: (**a**) macrophages differential cell counts were lower in HP compared to fIIP and CTD-ILD; (**b**) lymphocytes differential cell counts were higher in HP compared to fIIP and CTD-ILD. Values are shown as means and standard deviations. fIIP—fibrosing idiopathic interstitial pneumonia; CTD-ILD—interstitial lung diseases related to connective tissue disorder; HP—hypersensitive pneumonitis; **—*p* < 0.001; statistically significant *p*-values represent the exact two-tailed *p*-values of the Mann–Whitney test.

The results of lung function testing show forced vital capacity (FVC) and DL_CO_ as a percentage of predicted values (%PV). There was a significant difference (*p* < 0.05) in macrophages and lymphocytes cell counts between HPs and other groups. Macrophages differential cell counts were significantly lower in HP in comparison to fIIP and CTD-ILD, *p*- values were *p* = 0.0083 and *p* = 0.0032, respectively (Table 2, Figure 1a). Lymphocytes differential cell counts were significantly higher in HP in comparison to fIIP and CTD-ILD, *p*-values were *p* = 0.0070 and *p* = 0.0052, respectively (Table 2, Figure 1b).

**Figure 2:**
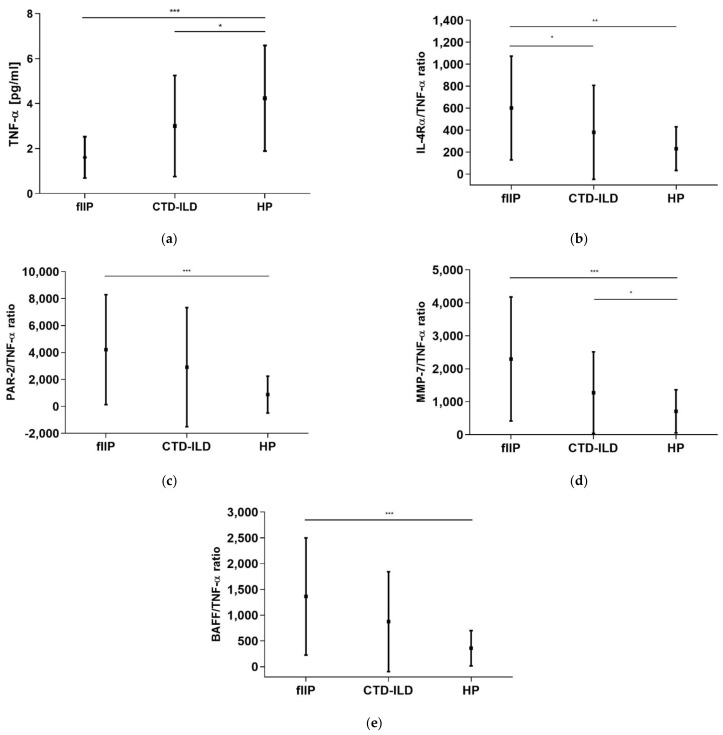
Significantly (**a**) higher levels of TNF-α were identified in HP compared to fIIP and CTD-ILD; (**b**) higher IL-4Rα/TNF-α ratios were identified in fIIP compared to CTD-ILD and HP; (**c**) lower PAR-2/TNF-α ratios were identified in HP compared to fIIP; (**d**) lower MMP-7/TNF-α ratios were identified in HPs compared to fIIP and CTD-ILD; (**e**) lower BAFF/TNF-α ratios were identified in HPs compared to fIIPs. Values are shown as means and standard deviations. TNF-α—tumor necrosis factor-α; IL-4Rα—interleukin-4 receptor α; MMP-7—matrix metalloproteinase-7; PAR-2—proteinase-activated receptor-2; BAFF—B cell-activating factor; fIIP—fibrosing idiopathic interstitial pneumonia; CTD-ILD—interstitial lung diseases related to connective tissue disorder; HP—hypersensitive pneumonitis; ***—*p* < 0.0001; **—*p* < 0.001; *—*p* < 0.001; statistically significant *p*-values represent the exact two-tailed *p*-values of the Mann–Whitney test.

When we compared concentrations of the measured analytes in the BALF of the IIP subgroups, there was a significant difference only in the concentration of TNF-α (Table 3, Figure 2a), which was significantly lower in fIIP compared to HP (*p* < 0.0001). CTD-ILD had significantly lower concentrations of TNF-α compared to HP (*p* = 0.0381) (Figure 2a). All possible ratios of the measured analytes were calculated. There were significant differences between the IL-4Rα/TNF-α, MMP-7/TNF-α, PAR-2/TNF-α, and BAFF/TNF-α ratios (Table 3, Figure 2b–e). For the IL-4Rα/TNF-α ratio, the *p*-values were 0.0074 and 0.0454 compared to fIIP vs. HP and fIIP vs. CTD-ILD, respectively (Table 3, Figure 2b). The PAR-2/TNF-α ratio was significantly higher in fIIP compared to HP (*p* = 0.0004) (Table 3, Figure 2c). For the MMP-7/TNF-α ratio, the *p*-values were < 0.0001 and 0.0237 compared to fIIP vs. HP and fIIP vs. CTD-ILD, respectively (Table 3, Figure 2d). The BAFF/TNF-α ratio was significantly higher in fIIP compared to HP (*p* = 0.0004) (Table 3, Figure 2e).

**Figure 3:**
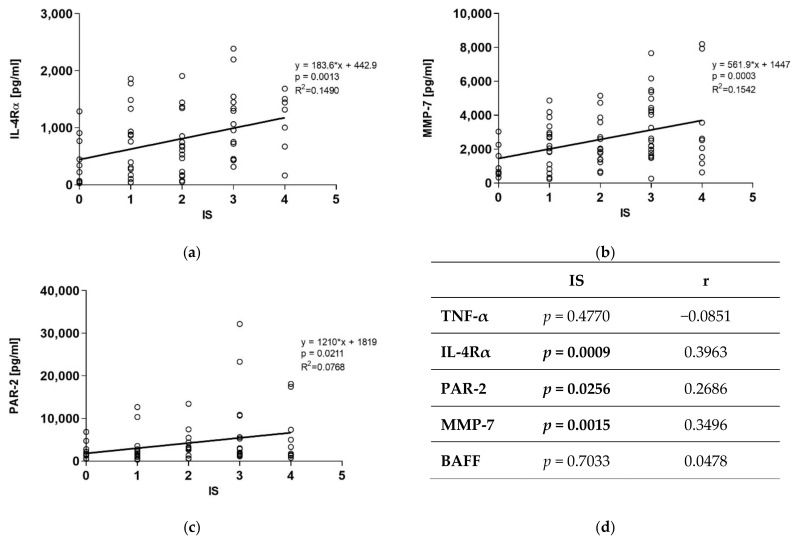
Linear regression and correlations between IS and individual BALF proteins concentrations in all pathologies tested together: (**a**) positive correlation between IS and BALF IL-4Rα concentration; (**b**) positive correlation between IS and BALF MMP-7 concentration; (**c**) positive correlation between IS and BALF PAR-2 concentration; (**d**) correlation between concentrations of analytes in BALF and IS; *p*-values represent the approximate two-tailed *p*-values of the Spearman’s correlation (statistically significant values in bold); IS—interstitial score; BALF—bronchoalveolar lavage fluid; TNF-α—tumor necrosis factor-α; IL-4Rα—interleukin 4 receptor α; MMP-7—matrix metalloproteinase-7; PAR-2—proteinase-activated receptor-2; BAFF—B cell-activating factor.

There was no significant correlation between AS and concentrations of TNF-α, IL- 4Rα, PAR-2, MMP-7, or BAFF in the BALF. On the contrary, we observed a significant correlation between IS and IL-4Rα, and PAR-2 and MMP-7, respectively (Figure 3). For all three analytes, the correlations were positive (Figure 3).

**Figure 4:**
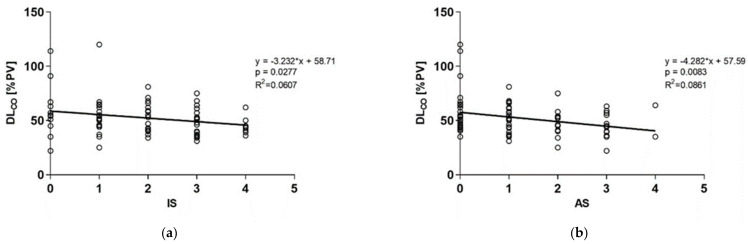
Correlations between HRCT scores and DL_CO_ in all pathologies tested together: (**a**) negative correlation between IS and DL_CO_; (**b**) negative correlation between AS and DL_CO_; IS—interstitial score; AS—alveolar score; DL_CO_—diffusing lung capacity for carbon monoxide; PV—predicted values.

When AS and IS were compared to the lung function tests and BALF differential cell counts, there were significant negative correlations for both scores with DL_CO_ (Table 4, Figure 4) and a there was significant positive correlation between IS and neutrophils (Table 4). We also correlated all measured analyte levels with lung functions. There was a significant negative correlation between MMP-7 and DL_CO_ (r = −0.2501, *p* = 0.0253).

## 4. Discussion

Bronchoscopy with bronchoalveolar lavage (BAL) is a relatively safe, minimally invasive, and well-tolerated procedure, which is performed in most patients with suspect IIP at the time of establishing a diagnosis [32,33]. Considering that BAL reaches the level of bronchial epithelium and alveoli, retrieved BALF has the potential to reflect ongoing immunopathogenic processes deep within the lungs. Based on previous research, there is an assumption of specific patterns of cytokines, chemokines, metalloproteinases, and other biologically active substances forming a profibrotic milieu [23,34,35,36,37]. Thus, BALF can provide samples for testing biomarker profiles for different types of IIP. As part of the innate immune response, AM constitute the first line of defense and provide pathogen recognition, initiation and resolution of lung inflammation, and repair of damaged tissue. According to the lung microenvironment, macrophages can operate as either pro- or anti-inflammatory or pro- or anti-fibrotic agents [38]. In our study, the lowest AM differential counts were found in HP, where the proinflammatory processes predominate. This is consistent with observations from Misharin et al. [39], who found elevated AM counts in the murine model of bleomycin—induced lung injury during the fibrotic phase. Another study suggested that the fibroproliferative processes in IPF might be driven by macrophages [40]. It was also found that higher neutrophil and eosinophil counts in IPF patients correlated with worse prognoses [41], which could be evaluated in the ongoing study.

TNF-α is an abundant mediator of inflammation with various biologic functions. It activates and recruits different inflammatory cells and plays a critical role in many chronic inflammatory diseases [19]. There is substantial evidence that TNF-α is also important in IPF progression [15]. Using an animal model, Miyazaki et al. discovered that chronic production of TNF-α leads to pulmonary lesions of varying severity, indicating that this cytokine is important in developing lung fibrosis [42]. High concentrations of TNF-α were found in the epithelial lung cells of IPF patients [10]. On the other hand, Fujita et al. showed that mice overexpressing TNF-α did not develop IPF after being induced with bleomycin or transforming growth factor (TGF)-β [15]. It seems that TNF-α may induce IPF as well as protect the lungs from developing IPF. The process that prevails is likely determined by a variety of host factors [11,15]. Pantelidis et al., in a study from 2001 [34], showed that polymorphisms of the TNF-α gene were not significantly associated with IPF. Assuming interactions among multiple alleles located in different genes on different chromosomes, they found an increased frequency of co-carriage of the interleukin (IL)-6 (intron 4G) allele on chromosome 7 and the TNF-RII(1960C) allele on chromosome 1 in patients with IPF. In another study, Chen et al. studied AM obtained from BALF samples taken from patients with HP as well as from healthy controls [12]. In patients with HP, they found increased concentrations of cytokines TNF-α, interleukin (IL)-18, as well as IL- 12 produced by AM cultured in RPMI medium, both without and after lipopolysaccharide stimulation (LPS). Their observations suggest that IL-12, IL-18, and TNF-α may be involved in the pathogenesis of HP. In our study, the concentrations of TNF-α in BALF were significantly lower in fIIP and CTD-ILD compared to HP. According to our previous [18,43] and present studies, we suggested that TNF-α levels are indicative of lung tissue inflammation, especially in the early phases of HP, rather than fibroproliferation. On the other hand, chronic stages of HP are characterized by more pronounced fibroproliferation rather than inflammation or granuloma formation, which is represented by an overexpression of IL-4Rα, as we previously described [44]. That is probably the reason why IL- 4Rα levels did not differ among the subgroups of IIP in our current study.

In our study, the levels of MMP7 did not significantly differ among the various pathologies tested, which corresponds with the research of K. Vuorinen et al. [13]. They found a significant negative correlation between MMP-7 and FVC. In our study, this correlation was also negative but not significant (data not shown). In a study published by Huh et al., there was also no significant difference between IIP and UIP or cryptogenic organizing pneumonia [45]. In addition, our results correspond with proMMP-7 (inactive form of MMP-7 from which it is produced) levels measured in the BALF of IPF patients, as previously described by Fujishima et al. [23]. They found negligible levels of proMMP- 7 in the BALF of healthy volunteers. They also measured concentrations of proMMP-7 in serum samples and found no significant difference in proMMP-7 levels between IPF patients and healthy volunteers. However, there are other studies that found statistically significant differences in serum MMP-7 in IPF compared with other IIP or healthy controls, which suggests that MMP-7 could be another tool to assist in the differential diagnosis of IPF [2,8,9]. Bauer et al. found that higher levels of MMP-7 in the serum of patients with IPF predict a higher risk of disease progression. Also, an increase in MMP- 7 levels and a decrease in FVC during longitudinal sampling were observed [9]. Rosas et al. correlated serum MMP-7 levels with lung functions [8]. They found a significant negative correlation between MMP-7 levels and FVC and DL_CO_, which corresponds with our observations. They also assessed gene expression of MMP-7 and MMP-1 in the lungs of patients with IPF and HP. Expression of both proteins can distinguish IPF from HP, which may help in clinical practice since nonfibrotic HP can be misdiagnosed as cellular NSIP and fibrotic HP can be indistinguishable from fibrotic NSIP or IPF [8]. From our study we assumed that, MMP-7 levels could correspond to the extent of the fibroproliferative process in the interstitium, which corresponds with reduced lung functions, especially DL_CO_ (r = −0.2501; *p* = 0.0253, data not shown). This assumption is supported by the positive correlation between MMP-7 and IS. However, BALF MMP-7 levels alone are not sufficient for a differential diagnosis of IIP. On the other hand, relative to the MMP7 to TNF-α ratio, MMP7-levels may serve to differentiate fIIP from CTD-ILD as well as from HP. 

Increased expression of PAR-2 was found in the lung tissue of patients with IPF compared to healthy volunteers. In fibroblasts, TGF-β, which is a crucial cytokine in IPF pathogenesis, strongly induced PAR-2 synthesis [14]. In our study, PAR-2 levels did not significantly differ among all IIP tested. In our previous study [46], BALF PAR-2 levels were significantly higher in HP compared to sarcoidosis (*p* < 0.0176). In addition, we found that the PAR-2/TNF-α and PAR-2/TGF-β ratios can distinguish HP from sarcoidosis [46]. We compared the PAR-2/TNF-α ratios in our present study and found a significant difference between the fIIP and HP group which, together with a positive correlation between PAR- 2 and IS, supports the presence of progressive fibrosis in lung tissue. At present, the PAR inhibitor OAc is in phase I clinical trials as a novel IPF treatment [47].

Significantly higher levels of BAFF were found in BALF samples of patients with IPF compared to healthy controls and in BALF samples of bleomycin-induced IPF in mice [48,49,50] In that experimental model, neutrophils were assessed to be the primary source of BAFF. Furthermore, in the murine IPF model, BAFF expression and lung fibrosis were IL- 1β- and IL-17A-dependent. Inhibition of BAFF led to IPF attenuation and a reduction in IL-1β levels [49,50]. Higher levels of BAFF were also found in the serum of patients with IPF and CTD-ILD in comparison with healthy controls [48,51,52,53]. Francois et al. showed that patients with systemic sclerosis (a CTD) and higher serum BAFF levels had a significantly higher incidence of pulmonary fibrosis and thus determined that BAFF was a potential therapeutic target [48]. In another work, Hamada et al. [51] found significantly higher serum BAFF levels in patients with CTD-ILD compared to patients with IPF. Additionally, they found a negative correlation between serum BAFF levels and lung function. These findings correspond with the work of Zhao et al. [52]. In our present work, the BAFF levels in BALF did not significantly differ among all the pathologies tested. In addition, there was no significant correlation between BAFF concentration and lung function, AS or IS. This might be due to the observation of Francois et al. who found elevated BAFF levels present in the BALF of IPF patients, especially when clinical exacerbation occurs [48]. 

We calculated the ratios of biomarkers similarly to previous studies [18,46,54] where they showed, in contrast with the single biomarker levels, statistically significant differences. We assumed this approach might better reflect different pathologies because they help to balance the disturbances in volume of retrieved BALF. In the present study, IL-4Rα/TNF-α ratio was significantly lower in CTD-ILD in comparison with fIIP and, thus, might distinguish these two pathologies better than single biomarker levels which, to the contrary, did not reach significant differences.

The positive correlation between IS and IL-4Rα supports our previous results, where we measured significantly higher concentrations of IL4-Rα in the BALF of patients with fIIP compared to controls [18]. IL-4Rα has been recognized as a key cytokine in the process of progressive fibrosis. Jakubzick et al. found that IL-4 and IL-13 receptor subunits were highly expressed in the SLB samples of patients with UIP and NSIP [55]. In addition, the expressions of IL-4Rα and IL-13Rα2 were defined as distinct foci in upper and lower SLB in patients with UIP compared to focal expression in patients with NSIP. In our previous research, which focused on cytokine gene polymorphisms and their influence on fibroproliferation, we found a significant correlation of the genotype at the promotor region of the IL-4 gene at positions (-590) (CT) and (-33) (CT) with IPF [56]. We assumed that the polymorphisms of IL-4, IL-4Rα, IL-1Rα, and IL-12 genes (genes of cytokines with regulatory activity) might influence the phenotype of IPF in the form of measurable changes in HRCT investigations [30]. In the present study, we showed that IL-4α concentrations positively correlated with IS, which further supports a possible role of IL-4Rα as a prognostic marker in fIIP.

We are aware of the limitations of our study. The main limitation was the relatively small number of patients involved, which prompted us to continue to enroll more patients with different types of IIP and to collect more data to support our findings and the potential practical application of our results for IIP. Another significant limitation was possible disturbances in BALF analytes concentrations related to differences in volumes of the retrieved BALF. In our previous studies, we dealt with different volumes of retrieved BALF by using BALF total protein measurements and/or by using analyte values in ratios. It is important to mention that even though we did not used total BALF protein measurements in this study, we still obtained results, which were in agreement with our previous studies and thus support our previous hypotheses.

## 5. Conclusions

In our study, we found positive correlations between IS and IL-4Rα, MMP-7, and PAR-2 levels and significant differences in biomarkers ratios in the BALF of patients with different types of IIP. Based on these results, we suggest that the complex levels of IL-4Rα and MMP-7, and possibly also PAR-2 in BALF might serve as useful biomarkers of fibroproliferation in IIP. In addition, we found a significant difference in TNF-α levels in the BALF of patients with various IIP; the highest levels were in patients with HP, reflecting the predominantly inflammatory nature of HP. We speculate that TNF-α might also serve as a useful tool in diagnosing IIP.

## Figures and Tables

**Table 1:** HRCT scoring system in IPF.

	Alveolar Score	Interstitial Score
0	0	0, no honey-combing
1	1–4%	1–4%, no honey-combing
2	5–24%	5–24%
3	15–49%	15–49%
4	50–74%	50–74%
5	75–100%	75–100%

HRCT—High Resolution Computer Tomography

**Table 2:** Demographic parameters, lung functions, and BALF differential cell counts of patients with different types of IIPs.

	fIIP	CTD-ILD	HP
Men/Women	18/6	7/13	13/23
Smokers/ex-smokers/non-smokers	2/17/5	2/6/12	3/16/17
Mean age [years]	67.9 (5.3)	61.7 (13.7)	64.1 (10.7)
AS	1.29 (1.12)	1.30 (1.08)	1.08 (1.18)
IS	2.25 (1.33)	1.90 (1.17)	1.75 (1.25)
FVC [%PV]	82.0 (16.7)	80.2 (23.3)	78.3 (22.2)
DL_CO_ [%PV]	53.5 (11.3)	54.5 (19.6)	50.6 (17.7)
BALF eosinophil counts [%]	7.8 (7.5)	5.2 (5.9)	7.3 (10.9)
BALF macrophage counts [%]	68.8 (15.6)	71.4 (22.9)	51.1 (25.0)
BALF lymphocyte counts [%]	13.2 (12.6)	12.9 (14.2)	33.5 (36.1)
BALF neutrophil counts [%]	10.5 (7.5)	8.2 (6.6)	6.9 (8.7)

Except for frequencies [numbers], results are shown as means and standard deviations in parentheses. AS—alveolar score; IS—interstitial score; FVC—forced vital capacity; PV—predicted values; DL_CO_—diffusion lung capacity for carbon monoxide; BALF—bronchoalveolar lavage fluid; fIIP—fibrosing idiopathic interstitial pneumonia; CTD-ILD—interstitial lung diseases related to connective tissue disorder; HP—hypersensitive pneumonitis.

**Table 3:** Measured analyte concentrations in different IIPs subgroups.

	fIIP	CTD-ILD	HP	*p*
TNF-α [pg/mL]	1.525 (1.07–1.83)	2.325 (1.41–4.365)	3.413 (2.911–4.793)	**<0.0001**
IL-4Rα [pg/mL]	758.6 (316.4–1318)	604.2 (286–763.45)	853.1 (170.4–1337.8)	0.5272
PAR-2 [pg/mL]	2040.8 (1287.78–9021.08)	2638.6 (1325.6–5476.9)	1881.1 (1262–3006,7)	0.6236
MMP-7 [pg/mL]	2567 (1834.5–3721.3)	1872 (1324.8–2597.5)	2120 (1040–3665)	0.4658
BAFF [pg/mL]	1030.0 (783.9–1410.1)	942.45 (689.6–1181.88)	1062.3 (693.3–1545.5)	0.6041
IL-4Rα/TNF-α	370.8 (269.4–801.9)	165.9 (114.5–548.5)	211.7 (40.2–411.6)	**0.0169**
PAR-2/TNF-α	2239.1 (944.8–6741.2)	1005.9 (467.9–3668.8)	487.2 (285.7–933.7)	**0.0016**
MMP-7/TNF-α	1590.0 (1191.0–2872.1)	979.1 (359.3–1470.8)	478.0 (240.9–976.7)	**<0.0001**
BAFF/TNF-α	909.7 (382.9–2340.2)	441.8 (236.3–911.6)	249.7 (120.7–476.0)	**0.0013**

Results are shown as means and standard deviations in parentheses; *p*-values represent the approximate two-tailed *p*-values of the Kruskal–Wallis test when comparing all groups (statistically significant values in bold); TNF-α—tumor necrosis factor-α; IL-4Rα—interleukin-4 receptor α; MMP-7—matrix metalloproteinase-7; PAR-2—proteinase-activated receptor-2; BAFF—B cell-activating factor; fIIP—fibrosing idiopathic interstitial pneumonia; CTD-ILD—interstitial lung diseases related to connective tissue disorder; HP—hypersensitive pneumonitis.

**Table 4:** Significant correlations between HRCT scores and neutrophils differential cell counts and DL_CO_.

	IS	r
neutrophils	*p* = 0.0051	0.3101
DL_CO_	*p* = 0.0400	−0.2301
	**AS**	
DL_CO_	*p* = 0.0164	−0.2675

DL_CO_—diffusion lung capacity for carbon monoxide; IS—interstitial score, AS—alveolar score; r—Spearman correlation coefficient; statistically significant *p*-values represent the approximate two-tailed *p*-values of the Spearman’s correlation.

## Data Availability

The data presented in this study are available on request from the corresponding author.
